# Michel's Process for External Tumors

**Published:** 1871-08

**Authors:** 


### ARTICLE IX.

### *Michel's Process for External Tumors.*

*The Lancet* gives a brief notice of the plan for removing tumors, purchased by Mr. Bell of M. Michel. In reply to some inquiries that have been made, the following details are added:

The preparation used in all cases where the tumor can be reached externally is made in the following way. Asbestos, as soft and free from grit as possible, is reduced by rubbing between the hands to the finest possible fleecy powder. It is then ground thoroughly with three times its own weight of strong sulphuric acid ($SO_3HO$), and worked into the desired shape with a silver or gold spatula. Mr. Bell remarks that the practical working of this powerful but simple escharotic is very remarkable; it destroys rapidly and completely whatever tissues lie directly beneath it, and in doing so neither causes the extreme pain which might naturally be expected, nor the local inflammation and general feverish symptoms which render similar applications undesirable.

In its application it is requisite that the patient should be so placed that the surface of the tumor is level. The adjoining parts of the skin are to be protected by a broad circle of collodion and a thick pad of soft linen. Nearly the whole surface of the tumor is to be then covered with a layer of asbestos and acid, the thickness of the latter being about half an inch for a tumor the size of a hen's egg; and to insure that the contact is uniform, the surface of the asbestos is to be covered with sovereigns. The skin beneath the mass is rapidly destroyed, a very narrow white circle marking the separation between the dead and living skin. The pulse remains quiet. The patient complains of considerable pain whilst the skin is being penetrated; it is not unbearable, however, and becomes much lessened after the first twenty minutes. Soon a slight oozing appears at some point from beneath the mass, the careful sopping up of which requires the undivided attention of the operator or experienced nurse for some hours. The length of time required for the destruction of a tumor varies with its size, its nearness to the surface, and the capability of the special tissue to resist the action of the mass; but in one the size of a hen's egg the first application may be left on from 8 A. M. till 10 P. M., when it may be removed; made up afresh to half the size and reapplied for the night, being finally removed on the following morning. The wound should appear dry, and the darker and more depressed it is the better.

The healing process then alone requires to be attended to, and this divided by Mr. Bell into (1) the stage of quiescence, (2) the suppurative stage, and (3) the healing stage, for the treatment of each of which minute directions are given. Mr. Bell is quite aware that this plan of treatment will not prevent the return of malignant disease, but he claims for it the following advantages:—1. That the shock of a surgical operation is entirely avoided. 2. That besides the tumor very little of the surrounding parts are injured or removed. And 3. That a malignant tumor when removed by means of the escharotic is not so rapidly recurrent as when it is excised.

